# Comparison of fecal and cecal microbiotas reveals qualitative similarities but quantitative differences

**DOI:** 10.1186/s12866-015-0388-6

**Published:** 2015-02-27

**Authors:** Dragana Stanley, Mark S Geier, Honglei Chen, Robert J Hughes, Robert J Moore

**Affiliations:** Central Queensland University, School of Medical and Applied Sciences, Bruce Highway, Rockhampton, QLD 4702 Australia; Australian Animal Health Laboratory, CSIRO Animal, Food and Health Sciences, Geelong, VIC 3220 Australia; RMIT University, Poultry Cooperative Research Centre, University of New England Armidale, New South Wales, 2315 Australia; South Australian Research and Development Institute, Pig and Poultry Production Institute, Roseworthy, South Australia 5371 Australia; The University of Adelaide, School of Animal and Veterinary Sciences Roseworthy, Roseworthy, South Australia 5371 Australia; University of South Australia, Research Office, Adelaide, South Australia 5001 Australia; RMIT University, Biotechnology and Ecological Biology, School of Applied Sciences, Bundoora, VIC 3083 Australia

**Keywords:** Intestinal, Microbiota, Chicken, Fecal, Cecal

## Abstract

**Background:**

The majority of chicken microbiota studies have used the ceca as a sampling site due to the specific role of ceca in chicken productivity, health and wellbeing. However, sampling from ceca and other gastrointestinal tract sections requires the bird to be sacrificed. In contrast, fecal sampling does not require sacrifice and thus allows the same bird to be sampled repeatedly over time. This is a more meaningful and preferred way of sampling as the same animals can be monitored and tracked for temporal studies. The commonly used practice of selecting a subset of birds at each time-point for sacrifice and sampling introduces added variability due to the known animal to animal variation in microbiota.

**Results:**

Cecal samples and fecal samples via cloacal swab were collected from 163 birds across 3 replicate trials. DNA was extracted and 16S rRNA gene sequences amplified and pyrosequenced to determine and compare the phylogenetic profile of the microbiota within each sample. The fecal and cecal samples were investigated to determine to what extent the microbiota found in fecal samples represented the microbiota of the ceca.

It was found that 88.55% of all operational taxonomic units (OTUs), containing 99.25% of all sequences, were shared between the two sample types, with OTUs unique for each sample type found to be very rare. There was a positive correlation between cecal and fecal abundance in the shared sequences, however the two communities differed significantly in community structure, represented as either alpha or beta diversity. The microbial populations present within the paired ceca of individual birds were also compared and shown to be similar.

**Conclusions:**

Fecal sample analysis captures a large percentage of the microbial diversity present in the ceca. However, the qualitative similarities in OTU presence are not a good representation of the proportions of OTUs within the microbiota from each sampling site. The fecal microbiota is qualitatively similar to cecal microbiota but quantitatively different. Fecal samples can be effectively used to detect some shifts and responses of cecal microbiota.

## Background

The avian ceca generally have a more important role in digestion than the cecum in most mammals. Avian ceca are usually finger-shaped blind pouches, presenting as lateral extensions at the junction of the small and large intestine and are commonly present in pairs. In birds the ceca vary considerably in size and morphology; ranging from very long, such as in most domestic poultry, to completely absent, such as in pigeons and parrots [1]. In the chicken they reach 16–18 cm long in adult birds. It is a multi-purpose organ vital to the bird’s physiology; a complex system inhabited by a very dense microbial community that converts the cecal pouches into fermentation powerhouses. Members of the cecal microbiota have the ability to digest cellulose, starch and other resistant polysaccharides [1,2]. Ceca are not only a major site of water absorption [1,3] but are also a site of nutrient transport and absorption [4].

Recent advances in culture-free technologies for microbiota characterization have facilitated an increasing number of studies investigating changes in chicken microbiota within the gastrointestinal tract (GIT) following manipulation of feed, environment, or health. It has been documented that chicken microbiota responds to changes in feed [5-7], litter composition [8], antibiotics [9] and probiotic addition to feed [10,11], disease [12,13] and stress [14,15]. Recently the use of next generation sequencing techniques to study microbiota composition has been extended beyond phylogenetic analysis to also include functional analysis using metagenomics of whole cecal microbiotas [16].

The important biological role of the cecum has resulted in it being a major focus of research into the influences of chicken microbiota on bird health and productivity [13,17-19]. The ceca sample microbes from both descending and ascending microbiota via normal peristalsis and retrograde gut movements [20,21]. The cecal content is emptied several times per day into the gut lumen and then regrows to fill the ceca. This cyclic emptying of the ceca means that elements of the fecal microbiota must be directly derived from the ceca. We were interested to examine the relationship between the population structure of cecal and fecal microbiotas to determine if fecal sampling via cloacal swabbing is an effective proxy for cecal sampling. Cloacal sampling, which is effectively a reliable and fast method of collecting fresh fecal samples from a specific bird, has the great advantage that it is easy to obtain a series of samples from the one bird over time, unlike the case with cecal samples where it is usual to sacrifice the bird to recover a sample and so only a single time-point snapshot can be obtained for any one bird. This has meant that temporal studies of cecal microbiota have had to rely on population sampling with different birds studied at each time point. The analysis of such data is complicated by the large inter-bird variation in microbiota structure that has recently been documented [22]. If the relationship between fecal and cecal samples was understood and consistent then fecal samples could be used to determine at least some elements of the microbiota present in the ceca.

The fecal and cecal microbiota profiles of mice have been compared using a gel based analysis method and no obvious correlations were noted [23] however the biology of the chicken ceca is very different to that of the mouse and so it is likely that this study gives little indication of the relationship of the two sites in chickens. Sekelja *et al.* [24] used conventional sequencing of cloned 16S fragments to investigate the variability of chicken fecal microbiota and how this was related to emptying of different regions of the GIT. Here we report a large chicken study that used next generation sequencing technology to characterize GIT microbiota. The study builds on previously reported work that analyzed the cecal microbiota of birds in a series of production efficiency trials [22]. Here we analyse further samples and extend the microbiota analysis to generate a statistically powerful data set to compare fecal and cecal sample pairs from 163 birds to determine the level and nature of similarity in microbiota structure between the two sample sites. The relationship between the microbiota in the two cecal compartments within each bird was also investigated.

## Results

### Cecal microbiota is richer in OTUs and has fewer dominant OTUs compared to fecal microbiota

A total of 1,770,812 sequences were obtained from the 326 samples included in this analysis (163 birds with adequate recovery of sequences from both cecal and fecal samples). After quality trimming and chimera checking 1,076,820 sequences remained with a minimum length of 324 nucleotides, a maximum length of 600, and an average read length of 507 nucleotides. There were on average over 3,300 sequences per sample.

**Figure 1:**
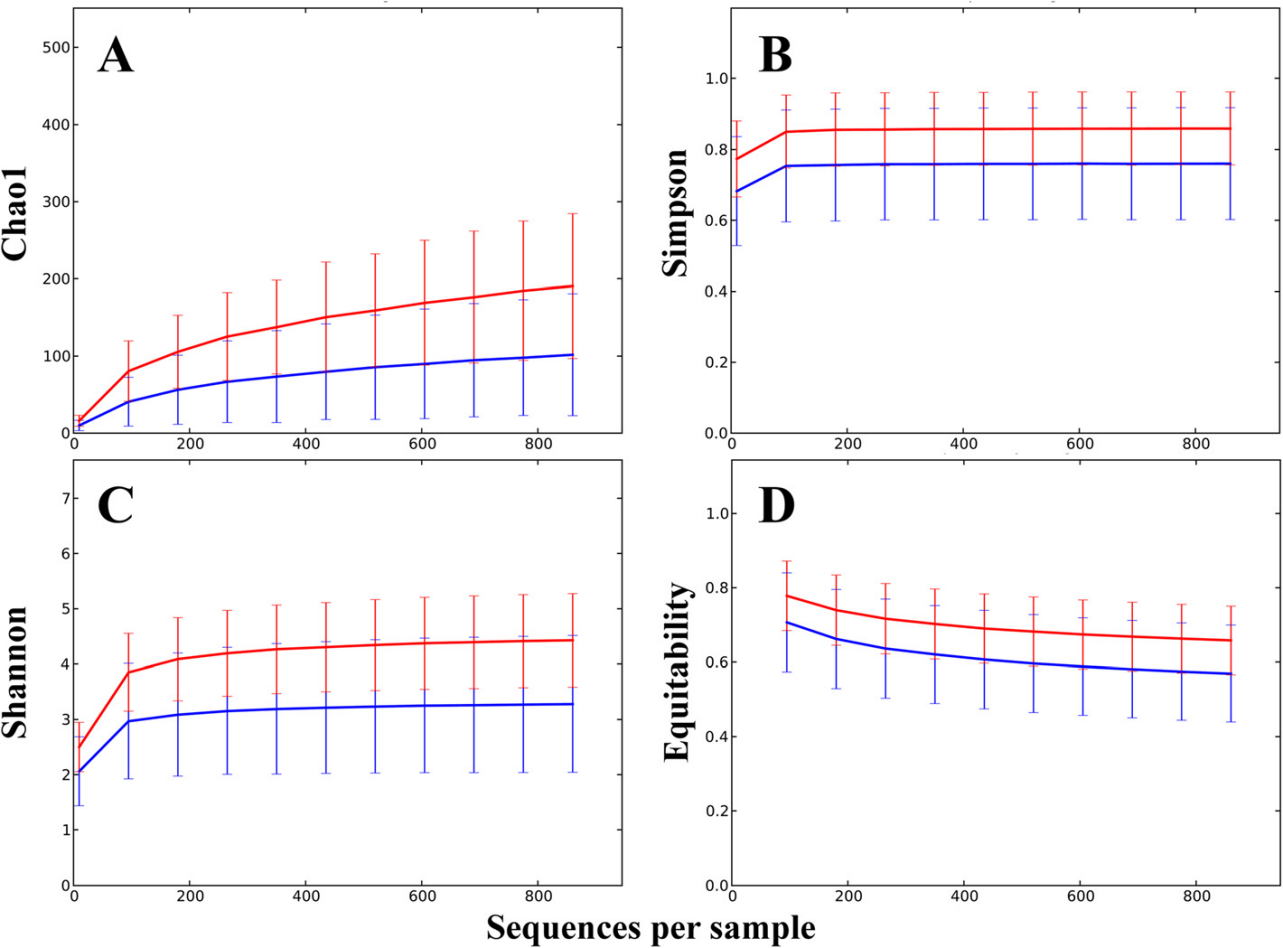
**Alpha diversity is significantly different between fecal and cecal microbiota.** Alpha diversity indicators of cecal (red) and fecal (blue) samples all showed significant (p < 1e^−5^) difference based on 1e^5^ Monte Carlo permutations. The indices plotted in the four panels are; **A**, Chao 1; **B**, Simpson; **C**, Shannon; and **D**, Equitability.

A number of alpha diversity measures were inspected to compare within sample diversity of cecal and fecal samples. All of the diversity indicators showed statistically significant differences, all with P < 1e^−5^, the lowest possible p-value based on the 1e^5^ Monte Carlo permutations used (Qiime). Both non-phylogenetic estimator Chao1 (Figure 1A) and phylogenetic diversity (PD) indicator whole tree analysis showed higher diversity in cecal samples relative to fecal samples. Cecal richness, based on number of observed species, was significantly higher than richness in fecal communities. The singles and doubles estimator suggested more rare OTUs in cecal samples. Simpson’s evenness (Figure 1B) and Strong’s dominance index confirmed that fecal samples have more dominant OTUs. Shannon entropy (Figure 1C) and the Equitability indices (Figure 1D) showed cecal phylotypes as more evenly distributed within samples.

**Figure 2:**
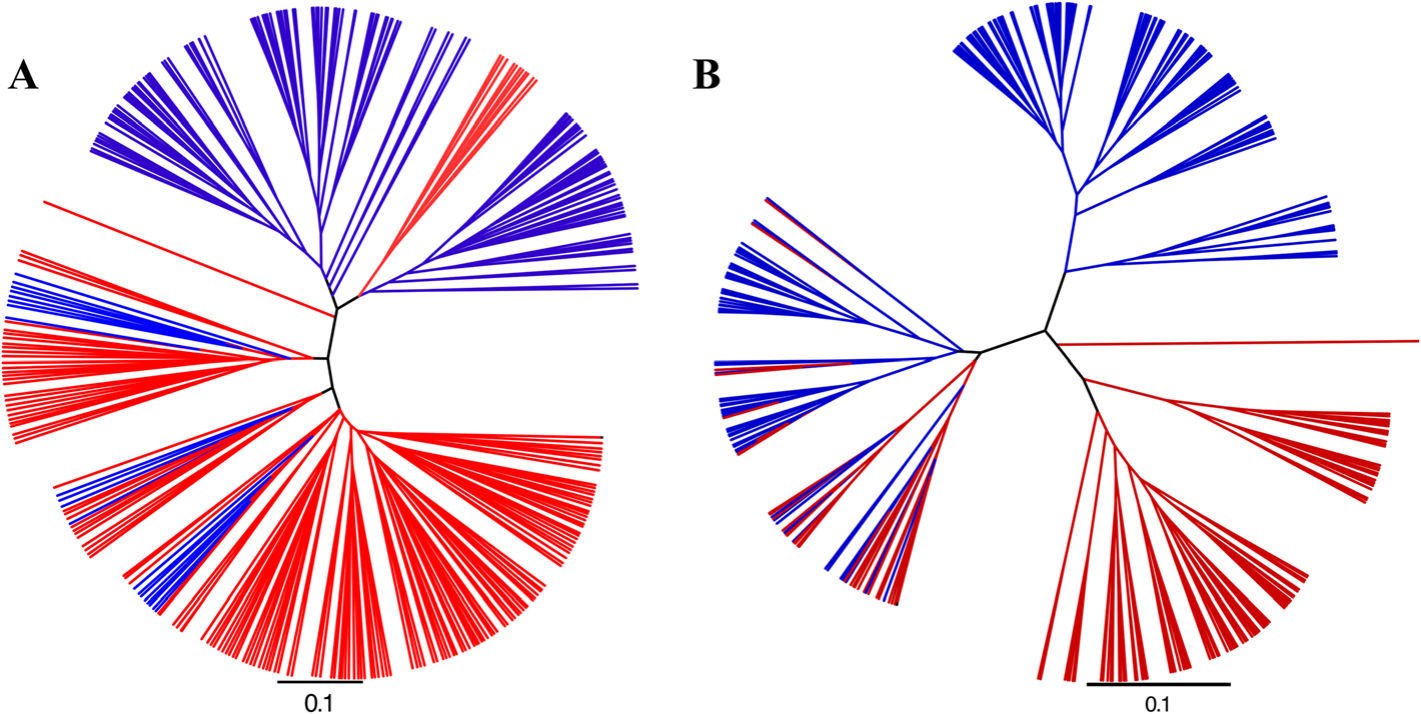
**UniFrac distance shows microbiota differences between fecal and cecal origins.** A tree representing cecal (red) and fecal (blue) samples. The sample clustering is based on jacknifed weighted **(A)** and unweighted **(B)** UniFrac. Tree files were generated in Qiime and visualised in FigTree. The two communities were significantly (p < 1e^−5^) different based on both weighted and unweighted UniFrac and ADONIS statistics using 1e^5^ permutations.

A range of beta diversity measures were inspected (Jaccard, Bray Curtis, Canberra, Chord, Euclidean, Manhattan, Pearson, Soergel,) and, similar to alpha diversity, they indicated significant differences between the fecal and cecal samples. Unweighted and weighted UniFrac (Figure 2) both showed differences in microbial communities with ADONIS statistics P-values lower than P < 1e^−5^, the lowest possible P-value based on the 1e^5^ permutations. Separation in both weighted and unweighted UniFrac indicated that the microbiota profiles group on origin (fecal or cecal) based on presence/absence as well as abundance.

### Members of cecal and fecal microbiota

**Figure 3:**
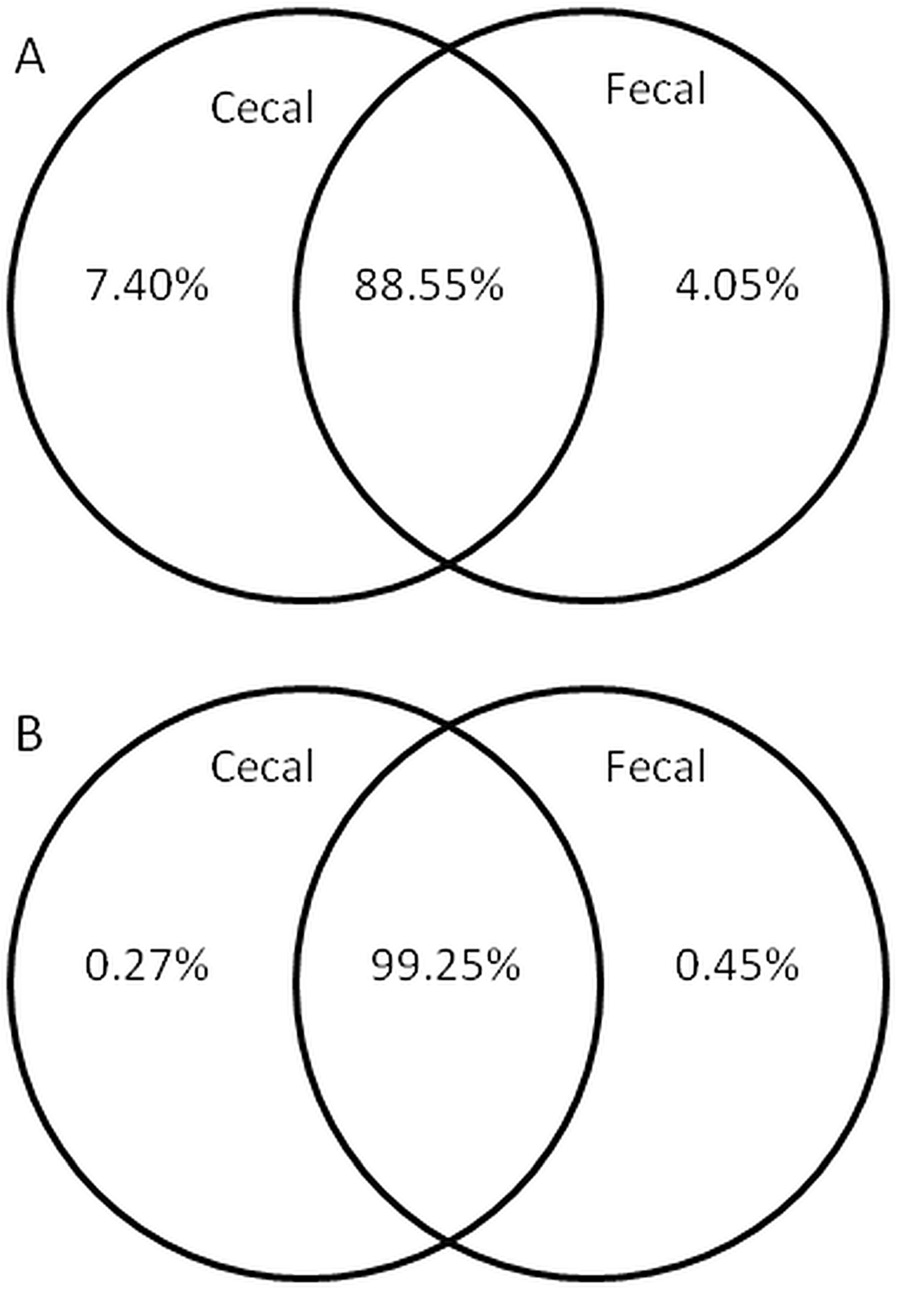
**Venn diagrams showing percentage of shared OTUs.** Diagrams are show the percentage of shared OTUs **(A)** and the percentage of sequence reads that they represented **(B)** of cecal and fecal origin at 3% divergence. 88.55% of all OTUs are shared between the sections and those OTUs represented 99.25% of all sequences.

Most of the OTUs were present in both cecal and fecal samples (Figure 3). The shared OTUs, represented by 88.55% of all OTUs, accounted for 99.25% of all sequences. The 7.4% of OTUs that were exclusive to cecal samples comprised only 0.27% of all sequences, indicating that they are mostly rare, low abundance OTUs. Fewer OTUs were exclusive to fecal samples (4.05%), however they made up a greater proportion of all sequences (0.45%) than the cecum exclusive OTUs. OTUs present only in cecum were all, with the exception of one OTU, unknown and uncultured relatives of *Bacteroides fragilis, Faecalibacterium prausnitzii,* and a number of unknown *Lactobacillus* and clostridia. On the other hand, 27% of OTUs exclusive to fecal origin shared sequence similarity higher than 97% to known type strains of bacteria not commonly found in chicken cecum such as *Streptococcus minor, Vagococcus fluvialis, Streptococcus henryi, Staphylococcus gallinarum* and *Staphylococcus aureus,* all with 100% sequence identity to type strains.

Tables 1 and 2 show the 20 most abundant OTUs, based on sequence counts, from cecal and cloacal samples respectively, classified using EzTaxon to their nearest culturable isolate. The most abundant OTU in chicken cecum, across the 3 trials, was 99.6% identical to a type strain of *Bacteroides fragilis* and represented 14.1% of all cecal sequences based on the 100-times rarefied data (Table 1). This OTU was the most abundant only in trial 1 with 20.3%, third most abundant in trial 2 (8%) and second most abundant in trial 3 with 14.4% of total sequences. The next 4 most abundant OTUs were confidently (>99% similarity) classified as *Lactobacillus crispatus*, *Lactobacillus johnsonii*, *Lactobacillus salivarius* and *Lactobacillus reuteri*. There were 3 more OTUs in the cecal top 20 classified with sequence similarity >97% to *Lactobacillus helveticus*, *Lactobacillus vaginalis* and *Parabacteroides distasonis*. The remaining OTUs, except for *Lactobacillus helveticus* (96.6%) were of unknown species with sequence similarity to the closest known isolates in the range of 91.9-78.3%. Cecal microbiota contained more unknown OTUs (similarity to known isolate <97%) than the fecal microbiota; those OTUs comprised 47% of cecal microbiota as opposed to 33.2% in fecal samples, based on total cecal and total fecal sequences.

**Table 1:** **The 20 most abundant OTUs in chicken cecum**

**Closest culturable isolate**	**Isolate accession number**	**% similarity**	**% in cecal**	**% in fecal**	**OTU ID**
*Bacteroides fragilis*	CR626927	99.62	14.1	1.2	4
*Lactobacillus crispatus*	Y17362	99.81	11.3	15.4	1
*Lactobacillus johnsonii*	ACGR01000047	99.61	9.6	12.2	2
*Lactobacillus salivarius*	AF089108	99.43	5.4	12.7	3
*Lactobacillus reuteri*	AP007281	99.40	4.3	5.6	5
*Acholeplasma palmae*	L33734	79.53	3.9	0.5	8
*Lactobacillus helveticus*	ACLM01000202	97.62	3.5	8.6	133
*Butyricicoccus pullicaecorum*	EU410376	82.18	3.4	0.2	9
*Faecalibacterium prausnitzii*	AJ413954	94.71	2.3	0.3	12
*Lactobacillus vaginalis*	AF243177	99.60	1.3	0.8	10
*Lactobacillus helveticus*	ACLM01000202	96.67	1.2	2.7	874
*Pontibacillus litoralis*	EU583724	78.46	0.9	0.2	13
*Exiguobacterium acetylicum*	X70313	78.33	0.9	0.1	14
*Ruminococcus albus*	L76598	82.62	0.9	0.1	21
*Ruminococcus albus*	L76598	89.88	0.8	0.1	16
*Parabacteroides distasonis*	CP000140	97.31	0.8	0.0	18
*Ruminococcus flavefaciens*	X83430	86.85	0.8	0.1	15
*Clostridium cellobioparum*	X71856	83.27	0.7	0.1	17
*Clostridium termitidis*	FR733680	80.37	0.7	0.1	23
*Clostridium symbiosum*	M59112	91.97	0.7	0.1	25

The representative sequences for all abundant OTUs are publically available in EMBL database with OTU ID as identifier with accession numbers HG810851- HG810882.

**Table 2:** **The 20 most abundant OTUs in chicken feces**

**Closest culturable isolate**	**Isolate accession number**	**% similarity**	**% in cecal**	**% in fecal**	**OTU ID**
*Lactobacillus crispatus*	Y17362	99.81	11.3	15.4	1
*Lactobacillus salivarius*	AF089108	99.43	5.4	12.7	3
*Lactobacillus johnsonii*	ACGR01000047	99.61	9.6	12.2	2
*Lactobacillus helveticus*	ACLM01000202	97.62	3.5	8.6	133
*Lactobacillus reuteri JC*	AP007281	99.40	4.3	5.6	5
*Clostridium perfringens*	CP000246	99.60	0.1	4.5	6
*Lactobacillus crispatus*	Y17362	91.98	0.3	2.9	2234
*Clostridium lituseburense*	M59107	96.91	0.4	2.8	7
*Lactobacillus salivarius*	AF089108	93.01	0.3	2.8	2200
*Lactobacillus helveticus*	ACLM01000202	96.67	1.2	2.7	874
*Candidatus Arthromitus sp.*	X80834	100.00	0.0	2.1	11
*Lactobacillus salivarius*	AF089108	94.44	0.1	2.1	1905
*Lactobacillus crispatus*	Y17362	94.50	0.1	1.2	1845
*Lactobacillus johnsonii*	ACGR01000047	89.41	0.2	1.2	2251
*Bacteroides fragilis*	CR626927	99.62	14.1	1.2	4
*Lactobacillus pontis*	AJ422032	92.01	0.1	1.0	2154
*Lactobacillus helveticus*	ACLM01000202	98.59	0.2	0.9	254
*Lactobacillus vaginalis*	AF243177	99.60	1.3	0.8	10
*Lactobacillus crispatus*	Y17362	93.60	0.1	0.7	1557
*Lactobacillus gallinarum*	AJ417737	90.77	0.1	0.6	1814

The representative sequences for all abundant OTUs are publically available in EMBL database with OTU ID as identifier with accession numbers HG810851- HG810882.

Fecal microbiota was dominated by *Lactobacillus* with the 5 most abundant OTUs making up 54.5% of all fecal sequences. The most abundant classified with >97% sequence similarity to type strains were *L. crispatus, L. salivarius, L. johnsonii, L. helveticus, L. reuteri* and *L. vaginalis*. Moreover, only 4 out of 20 most abundant OTUs were not Lactobacilli: *Bacteroides fragilis*, *Candidatus* Arthromitus, *Clostridium perfringens* and unknown clostridium similar to *C. lituseburense*. The remaining Lactobacilli OTUs, which showed sequence similarity to the closest culturable isolates of between 89.4 and 94.4%, most likely comprise a number of novel members of this genus. There were 406 OTUs out of 1282 total fecal and cecal, classified as most closely related to *Lactobacillus* strains, 351 of those with sequence similarity <95% to known culturable isolates.

Fecal samples contained *Clostridium perfringens* at an average level of 4.5%, the 6^th^ most abundant OTU classified, with 99.6% similarity to the type strain. However, cecal samples contained on average only 0.14% of this known chicken pathogen. The carriage of *C. perfringens* varied markedly across the 3 trials; in Trial 2 the fecal samples carried 12.8%, Trial 3 had 0.5% and none was detected in Trial 1. Although *C. perfringens* carriage was lower in the cecal samples the same relative trend between trials was noted with 0, 0.3 and 0.1% detected in trials 1–3 respectively.

### Low abundance fecal OTUs more closely correlate with cecal levels than high abundance OTUs

**Figure 4:**
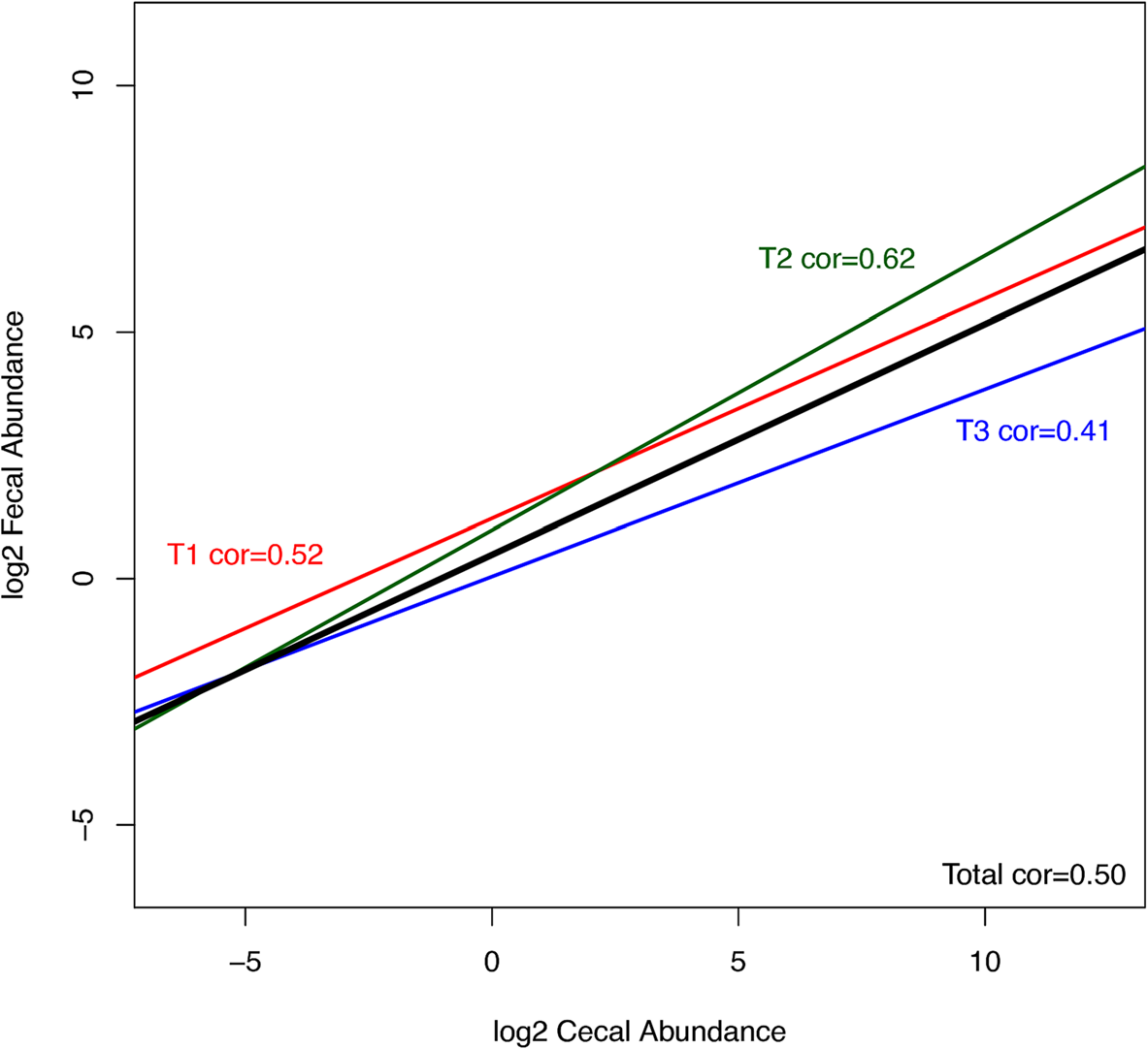
**Correlations between cecal and fecal abundance.** Line graph representing correlations between cecal and fecal abundance in trial 1 (red), trial 2 (green), trial 3 (blue) and complete set (black line). There is a reproducible positive correlation across the 3 trials. All individual OTUs present in both cecal and fecal sample for all 163 birds were used in calculation, however, due to a very high number they are not displayed in the plot.

**Figure 5:**
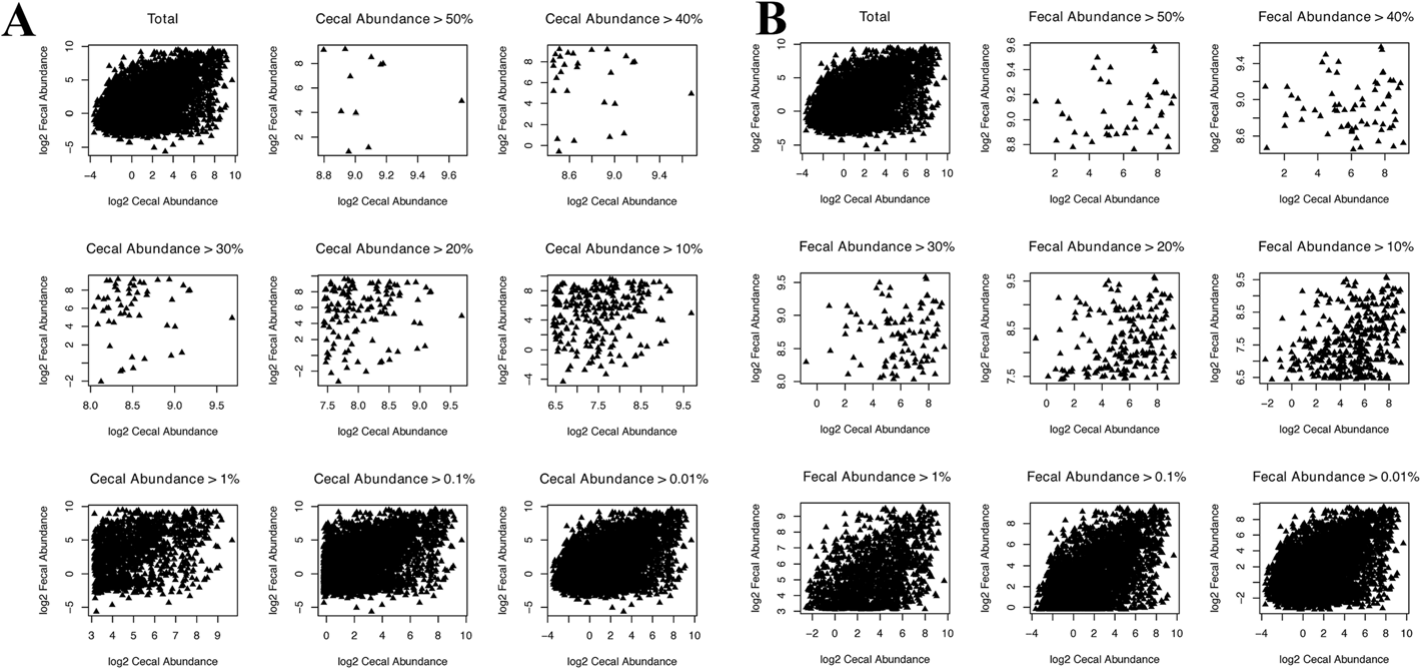
**Correlation plots for the different minimal cecal (A) and fecal (B) abundances.** Note that each shared OTU was plotted for all 163 birds and may be highly abundant in many of the birds, thus total percentages are not expected to add up to 100. For example, there were 11 birds that had OTUs with cecal abundance higher than 50% vs 43 birds that contained fecal OTUs with abundance over 50%.

**Figure 6:**
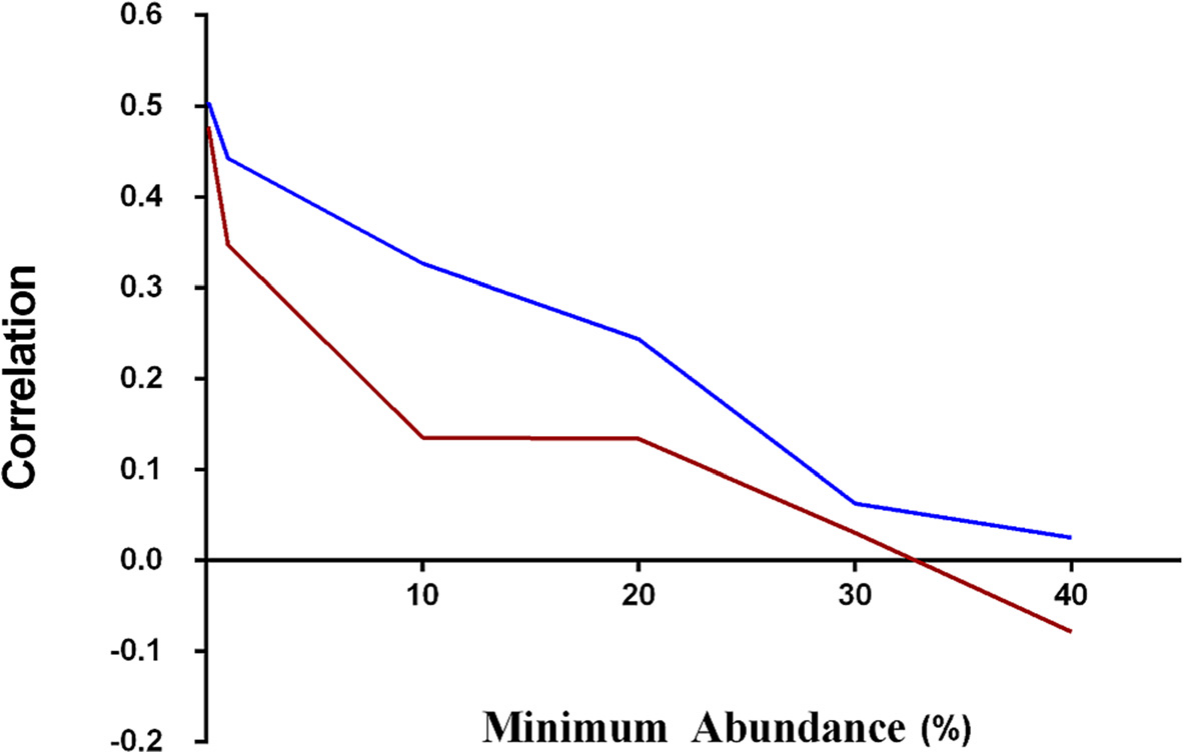
**Relationship between fecal/cecal correlation and levels of OTU abundance.** Correlations were plotted for both cecal (red) and fecal (blue) samples across the range of OTU abundances. There was linear relationship between correlation and minimum abundance (%) showing higher correlation for low abundance OTUs and a very low correlation for high abundance OTUs.

The main question we aimed to answer in this study was whether fecal samples could provide a reliable snapshot of cecal community structure. The high number of sequences and samples across the three trials provides sufficient statistical power to attempt to make these predictions. The three trials were inspected separately and as a whole set for correlations between fecal and cecal abundance of all shared OTUs present in both cecal and fecal samples across all of the birds. All 3 trials showed positive correlation between cecal and fecal abundances (Figure 4). To inspect if the level of correlation is influenced by abundance, i.e., if more abundant OTUs show better correlation, we inspected correlations for subsets of data with different cecal and separately fecal minimal abundance for all trials. Although we expected that more abundant OTUs would have higher cecal-fecal correlation, we found the opposite trend for both cecal and fecal abundances (Figures 4,5,6). Rare OTUs seem to be of similar low abundance in both cecal and fecal while higher abundance taxa tended to differ more. This is especially notable in the fecal dominant OTUs. OTUs comprising more than 50% of cecal sequences in one bird (Figure 5A) had a wide range of fecal abundances. The number of birds with cecal microbiota dominated by more than 50% was much lower than in fecal samples. Figure 6 simplifies the message from detailed Figure 5A and B; shared OTUs present in lower abundance correlated better, while correlation for more abundant OTUs was very low.

### The microbiota of cecal pairs are similar

**Figure 7:**
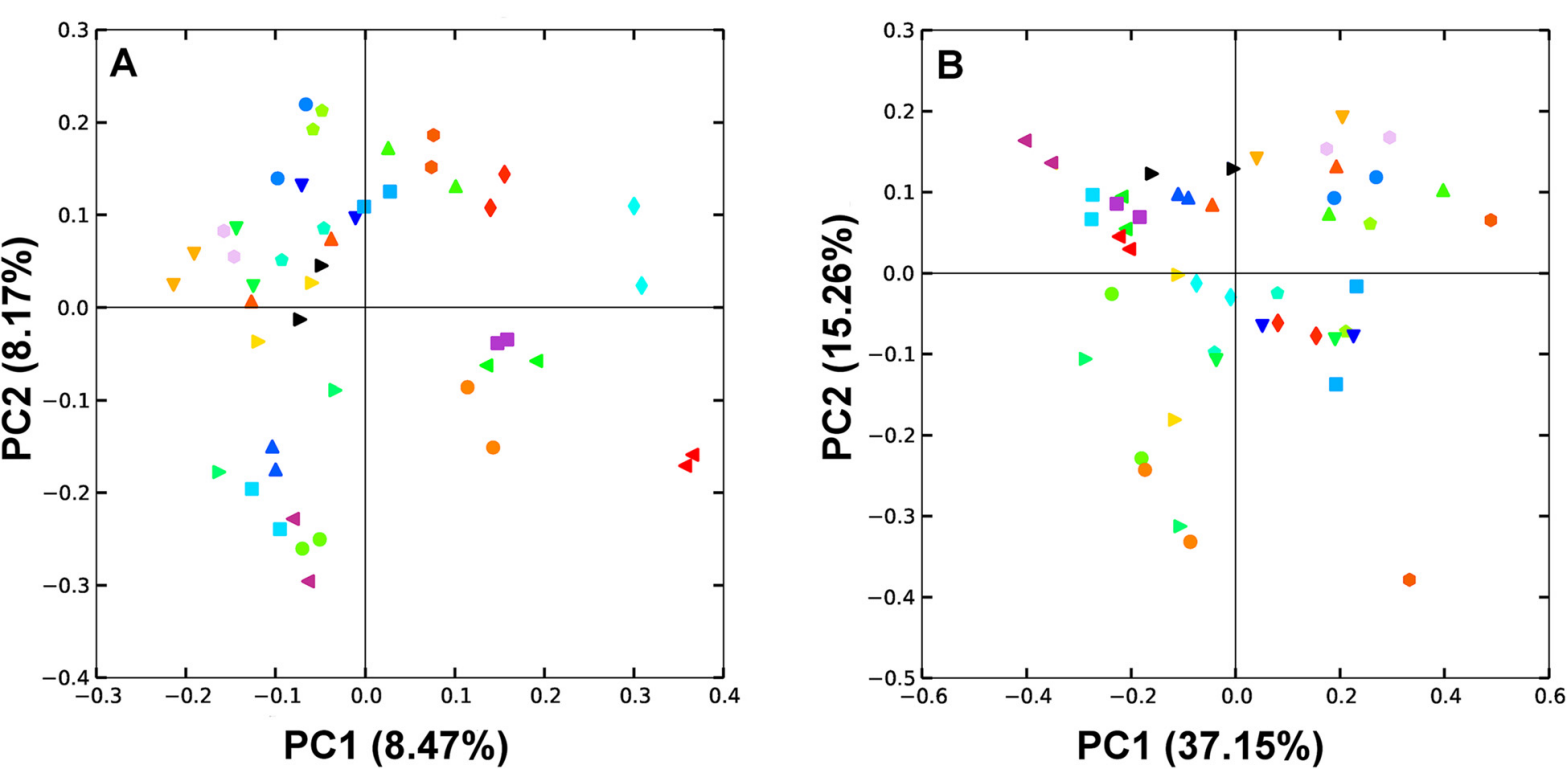
**Differences between left and right ceca samples from the same bird represented using UniFrac distance.** The unweighted **(A)** and weighted **(B)** UniFrac PCoA plots demonstrate that left and right ceca are harbouring similar bacterial communities. Left and right ceca from each bird are represented with the same unique symbol and colour combination.

The interpretation of cecal microbiota data could be influenced by sampling procedure if the pair of cecal pouches within a bird had radically different microbiota populations. To address this issue, in an independent analysis, each of the pair of ceca within 24 birds were sampled and analysed according to the same methods detailed for the main experiment. There was no statistically significant difference in alpha diversity between cecal pairs with Shannon, Simpson or Observed Species indices (P-values of 0.98, 0.77 and 0.56 respectively). Beta diversity was also unaffected by choice of ceca; Unweighted UniFrac differences using ADONIS statistics showed a P-value of 0.81 and Unweighted Unifrac P-value of 0.72. In Figure 7 it can be seen that the pairs of cecal samples from each bird tend to be closely related to each other and hence map close to each other in both the unweighted (Figure 7A) and weighted (Figure 7B) UniFrac plots.

## Discussion

Chicken cecal microbiota has been widely investigated due to the significant role of the cecum in heath, performance, and disease [1,2]. The first insights into cecal microbiota that suggested the complexity of the microbial populations came from culture-based studies. It has been suggested that the microbial diversity of chicken cecum has been altered with the common use of antibiotics in feed worldwide and that it has lost much of its natural chicken specialised microbiota and with it microbial potential and metabolic capabilities [1,25].

Ceca have been found to harbour microbiota capable of degrading cellulose and other indigestible carbohydrates and producing high amounts of beneficial metabolites such as short chain fatty acids (SCFA) [1]. Along the GIT the cecal community takes the most time to develop and mature [2] and it contains the highest microbial diversity within the GIT [13,26-29]. Although it was reported that differences between trials and flocks in chickens can be extensive, overall cecal composition identified in the present study agrees with previously published data. Lu *et al.* [28] found clostridia to dominate chicken cecum in their trial. In the present study 8 of the 20 most abundant OTUs, based on sequence counts and not adjusting for rRNA operon copy number, are most closely related to the Clostridiales order. However all of these had <95% sequence similarity to known culturable isolates, indicating that there is a high degree of cecal microbiota diversity that is yet to be revealed. In general the clostridia have a reputation as bad, undesirable members of the microbiota, based on pathogenic potential of strains of *C. perfringens*, *C. difficile*, *C. tetani* and a few others. However, this order also encompasses many beneficial bacteria such as cellulose and starch degraders; Clostridium clusters IV and XIV have been linked to prevention of inflammatory bowel disease (IBD) and maintenance of mucosal homeostasis [30,31] and clostridia protect from allergy and autoimmune disorders [32] in mice. Atarashi et. al. [32] reported the colonisation of germ free mice with a mix of Clostridium strains and also inspected mice enriched in *Clostridium* abundance. *Clostridium* strains promoted T_reg_ cell accumulation. Oral administration to conventionally grown mice, to increase *Clostridia* abundance, was beneficial for mice health, for example in inducing resistance to colitis by the means of suppressing weight loss, bleeding, colon shortening, edema and other symptoms of colitis allowing Clostridia supplemented mice to show mild, if any, symptoms. Chicken cecum is a major source of uncultured Clostridia that may represent enormous microbial potential.

The abundance of *Bacteroides, Lactobacillus* and *Clostridiales* in the cecum in the present study is in broad agreement with previous studies [18,19,26-29,33,34]. The benefits of *Lactobacillus* are well known, cecal samples in this study have shown high diversity in this genus and indicated potential for further probiotic research by estimating 351 potentially novel *Lactobacillus*-related species with similarity to the closest *Lactobacillus* database matches of <95%.

The sequence count data indicate that the most abundant OTU in the cecal microbiota was *B. fragilis* due to extreme values in one of the trials. *Bacteroides* are known to have beneficial effects on the host, being effective degraders of indigestible carbohydrates, especially cellulose and starch [35]. *B. fragilis* produces SCFA [36] and when colonising germ free animals aids T_reg_ differentiation and IL10 production [37]. Similarly another abundant phylotype related to *Faecalibacterium prausnitzii* belongs to a butyrate producing cluster [38]. Absence of *Faecalibacterium prausnitzii* is linked to Crohn’s disease [30] while cecal richness in *Ruminococcus* is in line with the previously reported [1] cellulose degrading potential [39] of the ceca.

Fecal microbiota was found to be dominated by *Lactobacillus*. This is consistent with previous studies [40]. The high abundance of *C. perfringens* was attributed to one trial with extreme values and *B. fragilis* was present but at much lower abundance than in the cecum. Surprisingly we identified an abundant fecal OTU, comprising 2.1% of fecal sequences, as 100% identical to Candidatus Arthromitus sp. LSFO1.94,LSFO2.94 (EzTaxon database type strain). Gong *et al.* [33] reported high abundance of Candidatus division Arthromitus in chicken jejunum and ileum mucosal samples, representing 34% and 28% of sequences in these regions respectively. They are found in gut microbiota of humans, chicken, rodents and fish, where they anchor to the intestinal epithelial cells in the ileum to act as immune system modulators [41]. They are major activators of T-cells [42,43], epithelial lymphocytes, [42,44] and IgA [45] and could thus also be important in chicken health and performance. Until recently Candidatus Arthromitus was the term used for two physiologically similar groups of segmented filamentous bacteria (SFB), one commonly found in the gut of arthropods and others commonly found in GIT of vertebrates [46]. Although there were striking similarities between the two groups, Thompson *et al.* [46] demonstrated them to be distinct and unrelated. Arthropod inhabiting filamentous bacteria have yet unknown function while the ones inhabiting GIT of vertebrates, including chicken, play absolutely critical roles in immune function of the host [47]. Thompson *et al.* suggested that GIT originating SFB form a monophyletic group in the Clostridiaceae based on 16S sequence analysis, are now renamed “Candidatus Savagella” [46]. However, arthropods specific filamentous bacteria were identified as members of a Lachnospiraceae Arthromitus cluster and should keep the name “Candidatus Arthromitus” [46]. The abundance of Candidatus Savagella in chicken fecal microbiota is not reproducible across the studies. In our own opinion its identification is influenced by the taxonomic databases and algorithms used and in many cases they fall under unknown and uncultured bacteria. It is possible that their role in chicken health, especially immunity is significant therefore more attention should be given to deeper taxonomic identification of significant OTUs in chicken studies.

Sekelja *et al.* [24] inspected the influence of other GIT sections on chicken fecal microbiota over 16 days. They detected massive temporal variations in fecal microbiota and different profiles to match different sections of GIT. They proposed that the major reason for the temporal variation was periodic emptying of different GIT sections. The emptying of different GIT areas may influence fecal profile but fecal analysis still remains a useful and powerful approach for microbiota studies in animals with proven success record in human and mammalian studies. It is possible that the timing of sampling and cecal emptying is the reason for the existence of a number of outlier birds with higher similarity between fecal and cecal communities. In an auxiliary study we found that there were strong similarities between the microbiota compositions of pairs of ceca from birds, indicating that the choice of ceca is unlikely to be a concern in sampling procedures. Perhaps a major lesson to be learnt is that it may be misleading to draw conclusions from just a few samples; a large number of samples will represent a range of different emptying events from different regions of the GIT and may allow a more complete and representative overall picture to emerge of microbiota composition.

In the present study we compared fecal and cecal microbiota across many birds and three independent trials in order to investigate the relationship between the two most commonly used and reported sample types for microbiota analysis in birds. We were particularly interested in determining the extent to which the fecal microbiota may reflect the content of the cecal microbiota. From this extensive sample set we can conclude that for the majority of OTUs (88.55%), comprising 99.25% of sequences, presence in fecal implies presence in cecal community.

Our results show the highest correlation between fecal and cecal samples within the rare biosphere species, the correlation analysis however, included only shared OTUs, but all OTUs that were not shared (i.e., present only in fecal or only in cecal samples) were also from the low abundance group. Therefore, high correlation among rare OTUs cannot be used to exactly predict cecal from fecal abundance since some rare OTUs are also likely not to be detected in the other community at all. The data shows that OTUs exclusive to cecum, which would be missed if fecal origin was sampled instead, were all of low abundance.

Based on the positive correlation for nearly all shared OTUs it is possible that the low abundance OTUs that appear to be unique to the cecal or to fecal samples would be identified in the opposite group if the depth of sequencing was higher as they are near the detection limit and the limits of the exclusion criteria used in quality filtering parameters to generate the data set. Although the alpha diversity plots indicated satisfactory sequence coverage an increase in sequencing depth may identify more shared OTUs but is also likely to detect additional rare OTUs. There is a positive overall correlation between cecal and fecal abundance as shown in Figure 4; this correlation is however negligible in the more dominant OTUs comprising over 10% of total sequences. This trend was found to be reproducible over the three trials that harbour very different microbial communities.

## Conclusions

Based on the findings of the present study, we anticipate that with enough sequencing depth, fecal samples can be used to reflect the presence and absence of the vast majority of the members of the cecal community. However, the two communities are generally very distinct based on alpha and beta diversity. Positive correlation cannot be used to accurately predict OTU counts in the low abundance part of the microbiota due to it also carrying the highest number of unshared OTUs, nor in high abundance OTUs where correlation is minimal. However, regardless of community structure differences, i.e., species showing different abundance, dominance and equitability, both cecal and fecal microbiota analyses are likely to accurately report if a treatment or condition has induced changes in microbiota. This conclusion is based on the high number of shared species that represent 99.25% of all community members, which would be part of the community response to treatment. Although control/treatment differences could be detected using either cecal or fecal samples, conclusions drawn from the separate analyses are likely to differ. Hence choice of sampling site remains critical in experimental design as fecal microbiota do not provide a complete indication of cecal community structure.

## Methods

### Chicken trials

The bird trials were performed as previously described [18]. Briefly, male Cobb 500 broiler chickens were reared on feed comprised of wheat, soybean, barley, canola, peas, meat, tallow, limestone and vitamin mix with free access to food and water. Birds were euthanized by cervical dislocation on day 25 and samples collected for microbial analysis. Three independent replicate trials were performed. Samples were collected from an average of 70 birds from each trial. Cloacal (fecal) swabs were taken with sterile cotton swab inserted 10–12 mm into the cloacal opening and gently rotated to collect a sample of the fecal material. Cecal contents were collected by opening the birds immediately after euthanasia, cutting off one cecum, and manually squeezing content into a sterile tube. From one trial both ceca were harvested from 20 birds and content of each taken as separate samples. The swabs and cecal content were snap frozen on dry-ice and transported to the laboratory for processing.

### Ethics statement

All animal work was conducted according to the national and international guidelines for animal welfare. The animal trials were approved and monitored by the Animal Ethics Committees of the University of Adelaide (Approval No. S-2010-080) and the Department of Primary Industries and Resources, South Australia (Approval No. 08/10).

### DNA preparation, sequencing, and data analysis

DNA was prepared as described before, following the method detailed in Yu and Morrison [48]. The primers used to PCR amplify the V1-V3 region of the 16S rRNA gene were (forward primer [49], 5′ AGAGTTTGATCCTGG 3′; reverse primer [50]), 5′ TTACCGCGGCTGCT 3′). Both primers also included 454 sequencing regions and the series of forward primers included bar-code sequences to allow multiplexing of samples in a sequencing pool. Pyrosequencing was performed using a Roche/454 FLX Genome Sequencer according to the manufacturer’s instructions. Sequences were analysed using PyroBayes [51], pintail chimera detection algorithm [52] and Qiime v1.6.0 [53]. Sequences were quality trimmed as described previously [48]. OTU picking was done at 3% divergence level, de-noising error-correction, abundance and amplicon estimation using the USEARCH algorithm [54,55]. OTUs represented with less than 10 sequences and present in less than 5 samples were filtered out of the analysis. Samples represented by fewer than 1000 quality trimmed and filtered sequences were removed from the analysis and to maintain a paired data design the other sample of the rejected fecal/cecal sample pair was also removed, resulting in a total of 326 sequenced samples, from 163 birds, across three independent trials (52, 54 and 57 birds for trial 1 to trial 3 respectively), each bird represented with both fecal and cecal sample. Normalization of OTU tables was done by performing multiple rarefactions 100 times and averaging counts using a custom Perl script. OTUs were matched to their closest culturable isolate using EzTaxon [56]. R statistical software was used to inspect the correlation between cecal and fecal samples. The amplicon sequence data is available at MG-RAST under accession number 4614960.3 (http://metagenomics.anl.gov/metagenomics.cgi?page=MetagenomeOverview&metagenome=4614960.3).
